# Associations of depressive and anxiety symptoms with non-suicidal self-injury and suicidal attempt among Chinese adolescents: The mediation role of sleep quality

**DOI:** 10.3389/fpsyt.2022.1018525

**Published:** 2022-12-22

**Authors:** Ting Jiao, Shuangshuang Guo, Yi Zhang, Yanqi Li, Xinyi Xie, Ying Ma, Ruoling Chen, Yizhen Yu, Jie Tang

**Affiliations:** ^1^Department of Preventive Medicine, School of Public Health, Guangzhou Medical University, Guangzhou, China; ^2^Department of Child Healthcare, Guangzhou Women and Children’s Medical Center, Guangzhou Medical University, Guangzhou, China; ^3^Faculty of Education, Health and Wellbeing, University of Wolverhampton, Wolverhampton, United Kingdom; ^4^Department of Maternal and Child Healthcare, School of Public Health, Tongji Medical College, Huazhong University of Science and Technology, Wuhan, China

**Keywords:** adolescents, anxiety, depressive symptoms, non-suicidal self-injury (NSSI), sleep quality, suicide attempt (SA)

## Abstract

**Background:**

Associations of depressive and anxiety symptoms with non-suicidal self-injury (NSSI) and suicide attempt (SA) are not well understood. We aimed to examine these associations among Chinese adolescents, and whether any potential association is mediated through sleep quality.

**Methods:**

We conducted a cross-sectional study among 1,771 (994 boys [56.1%] and 777 girls [43.9%], mean [SD] age was 12.9 [0.6] years) adolescents who participated in the baseline survey of the Chinese Adolescent Health Growth Cohort (CAHGC) study. Depressive symptoms, anxiety symptoms, NSSI, SA and sleep quality were measured by validated questionnaire. Logistic regression models were employed to estimate the associations of depression and anxiety with NSSI and SA. Mediation analyses were conducted to explore the mediate effect of sleep quality.

**Results:**

The 12-month prevalence of NSSI and SA was 17.1 and 8.3%, respectively. Depressive and anxiety symptoms were significantly associated with NSSI (the adjusted odds ratio [aOR] was 1.89 [95% CI 1.34–2.65] for depressive symptoms and 2.84 [95% CI 2.05–3.94] for anxiety symptoms) and SA (the aOR was 3.20 [95% CI 2.03–5.05] for depressive symptoms and 2.98 [95% CI 1.84–4.84] for anxiety symptoms). No significant gender differences were found in the associations. The mediation proportion of sleep quality on the association of depressive and anxiety symptoms with NSSI, as well as depressive and anxiety symptoms with SA were 21.1, 13.9, 13.6, and 14.7, respectively.

**Conclusion:**

Independent associations of depressive and anxiety symptoms with NSSI and SA were observed in Chinese adolescents, and there were no significant gender differences in the associations. Moreover, these associations were partially mediated through sleep quality. Targeted interventions for adolescents’ NSSI and SA should focus on those who have depressive and anxiety symptoms, and poor sleep quality.

## 1 Introduction

Non-suicidal self-injury (NSSI) and suicide attempt (SA) are major public concerns among adolescents worldwide (1), with a prevalence of 13.3–27.6% of NSSI and 3.3–5.9% of SA in children aged 12–15 years, varying across countries and between genders (2). SA and NSSI can be grouped into the overarching category of self-harm (3). The difference between NSSI and SA is the absence or presence of suicidal intent when self-harming, although some studies in the literature have methodologically decided not to make this distinction (4). The occurrence of NSSI and SA may be more common during and after COVID-19 pandemic due to COVID-19 related social distance and school lockdown policies, and isolation (5–7). Although NSSI and SA differ in frequency and purpose of behavior, both are the strongest and most consistent predictors of future suicidal behaviors. A lifetime history of NSSI is associated with a roughly fourfold increased likelihood of future SA (8). Up to 2% of patients admitted to hospitals for NSSI after 1 year and 7% after 9 years died by suicide (9). Therefore, it is critical to understand further their risk factors and the complex interplay between many of the recognized risk factors.

Previous studies indicated that multiple factors were associated with NSSI and SA, including mental health difficulties and sleep disturbance (1, 10, 11). Depressive and anxiety symptoms are the most common mental health difficulties in adolescents (12), which were considered to be significant risk factors for NSSI and SA (10, 13), although some previous studies suggested depressive and anxiety symptoms appear to diverge with respect to suicidality (13, 14). However, most studies were derived from western countries and from clinical populations (13, 15), with few studies conducted in general adolescent populations (16). Furthermore, a great body of studies has reported gender differences in epidemiology of depressive and anxiety symptoms, NSSI and SA in adolescents (1, 17), yet only a few studies have examined whether there are gender differences in the associations and drawn mixed conclusion (18, 19).

In addition to depressive and anxiety symptoms, many studies indicated that sleep problems were also associated with NSSI and SA (20, 21). Sleep problems, including short/long sleep duration, poor sleep quality, insomnia and frequent nightmares, often occur among adolescents due to sleep patterns change markedly at puberty. Theoretically, the association of depressive and anxiety symptoms with NSSI and SA may mediate through sleep problems, because both depressive and anxiety symptoms among adolescents could change biological and psychosocial status, which then associated with sleep problems (22). However, to our knowledge, no study has examined the role of sleep quality as a mediator in these associations, although one study examined the role of sleep quality as a mediator in the association of emotional and behavioral problems with suicidal ideation (23). Owing to the importance of NSSI and SA on adolescent health, addressing limitations in previous studies is essential to not just gain a fuller understanding of how depressive symptoms, anxiety symptoms, sleep quality, NSSI and SA interrelate, but also for policymaker developing to implement prevention and intervention of NSSI and SA.

The present study aimed to investigate the association of depressive and anxiety symptoms with NSSI and SA and whether there were gender differences in a Chinese general adolescent population. We also aimed to examine whether these associations were mediated through poor sleep quality.

## 2 Materials and methods

### 2.1 Study design, participants, and procedure

This study was based on the baseline survey data of Chinese Adolescent Health Growth Cohort (CAHGC) study, an ongoing cohort established across three cities (Hengyang in Hunan province, Shenzhen and Zhongshan in Guangdong Province) in China in 2020. The CAHGC aimed to examine the influencing factors of abnormal behaviors (mainly self-harm behaviors) and their developmental trajectory among Chinese adolescents. The study baseline survey started in 2021 when participants first completed a questionnaire on their profile, socioeconomic status, psychological status, behavior characteristics, and took physical examinations.

Since the onset of self-harm behaviors usually occurs in early adolescence, we recruited the students who were in seventh grade as the study population. A random cluster sampling method was used to select the participants. With the help of local administration in each study site, 11 schools (3 in Hengyang city, 2 in Shenzhen city, and 6 in Zhongshan city) were randomly selected for the CAHGC study. All the seventh-grade students in the selected schools were eligible to participate if they did not have severe mental disorders (e.g., moderate to severe depression, schizophrenia, and bipolar disorder) and severe physical diseases (e.g., heart disease, nephropathy, and diseases of the immune system) identified by the head teacher and/or healthcare physicians. A consent form was sent to 1,844 students from 42 classes in the selected schools to obtain their guardians’ written informed consent before participating in the baseline survey.

Trained investigators, comprised of teachers and postgraduates, conducted the baseline survey and they were available at each study site to clarify the participant’s possible confusion and questions about the structured questionnaire. Participants completed the survey in a single sitting while at school. Before the survey, participants were informed of the purpose and procedures of the study in detail and were required to complete the questionnaire independently within 45–60 min. Completeness of the questionnaire was reviewed by investigators. Clinical professionals from the local medical institutions then did physical examinations and collected blood samples. The CAHGC study was approved by the Institutional Review Board of Guangzhou Medical University (NO. 2021010002). The present study received ethics clearance from Guangzhou Medical University due to use of the de-identified data and followed the American Association for Public Opinion Research (AAPOR).

### 2.2 Instruments

#### 2.2.1 Measurement of sociodemographic profile, depression, anxiety, sleep quality, emotional management ability, and social support

##### 2.2.1.1 Measurement of sociodemographic profile

We used a custom-designed questionnaire to collect demographic, familial, and academic pressure variables, including regional areas (Hengyang, Shenzhen, or Zhongshan city), ethnicity (Han or others), age, gender (boy or girl), single-child family (yes or no), family structure (core/joint family, single parent family, or recombine/cross-generation family), educational level of the main caregiver (junior high school or below, senior high school, college or above), living environment (good, moderate or poor), and academic pressure (heavy or more, moderate or less).

##### 2.2.1.2 Depressive symptoms

Depressive symptoms were measured by using the Chinese version of Center for Epidemiological Studies Depression Scale (CESD) (24), which is widely used for depressive symptoms assessment among adolescents in China (25). The CESD comprises 9 items, among which 7 items assess negative symptoms (e.g., “I felt depressed” and “I walked slowly”) and 2 items measure positive affect (“I was happy” and “I enjoyed life”). Each item uses a four-point Likert-scale: 0, < 1 day per week; 1, 1–2 days per week; 2, 3–4 days per week; and 3; 5–7 days per week; thus, total scores on the CESD range from 0 to 27. The higher total scores indicate greater symptoms of depression. Participants were divided into two groups based on the cutoff point proposed by He (24). Participants with CESD scores between 10 and 27 were classified as having depressive symptoms and CESD scores between 0 and 9 were as no depressive symptoms (26). The CESD has been demonstrated to have a good internal consistency, with a Cronbach’s α coefficient of 0.88 in a previous study (27) and 0.83 in the present study.

##### 2.2.1.3 Anxiety symptoms

The Chinese version of Generalized Anxiety Disorder Assessment (GAD) was used to measure generalized anxiety symptoms (28). It consists of seven items using four-point Likert scale (0, < 1 day per week; 1, 1–2 days per week; 2, 3–4 days per week; and 3; 5–7 days per week). The total scores of the GAD range from 0 to 21. Participants were divided into two groups based on the total scores of the GAD: (1) scores between 5 and 21, defined as having anxiety symptoms; and (2) scores between 0 and 4, defined as not having anxiety symptoms (28). Previous studies have shown that the GAD has good reliability and validity, and the Cronbach’s α coefficient of the scale was 0.93–0.95 (29). In the present study, the Cronbach’s α coefficient was 0.92.

##### 2.2.1.4 Sleep quality

The Pittsburg Sleep Quality Index (PSQI) was used to assess sleep quality (30). This self-rating questionnaire includes seven components assessing subjective sleep quality, sleep latency, sleep duration, sleep efficiency, sleep disturbance, use of sleep medication, and daytime functioning. In scoring the PSQI, 7 component scores are derived, each scored 0 (no difficulty) to 3 (severe difficulty). The component scores are summed to produce a global score (ranging from 0 to 21). Higher scores indicate poorer sleep quality. Based on the cutoff point used in a previous study (31), participants with global scores between 6 and 21 were classified as having poor sleep quality. Participants with global scores between 0 and 5 were classified as having a good sleep quality. Previous study has demonstrated the PSQI has adequate reliability with Cronbach’s α coefficient was 0.87 among Chinese adolescents (32).

##### 2.2.1.5 Emotional management ability

A four-item subscale from the Emotional Intelligence Inventory was used to measure emotional management ability (33). This scale used four-point Likert responses: 1, always like this; 2, often like this; 3, rarely like this; and 4, never like this). Higher total scores represent greater emotional management ability. The Cronbach’s α coefficient of this subscale was 0.78 in previous study (34) and was 0.84 in present study.

##### 2.2.1.6 Social support

Social support was assessed by the 17-item Adolescent Social Support Scale (35), which has five-point Likert-type responses for each item: 1, strongly agree; 2, agree; 3, neutral; 4, somewhat disagree; and 5, strongly disagree. The scale scores range from 17 to 85, higher total scores represent greater social support. The Cronbach’s α coefficient of the scale was 0.93 in previous study (34) and 0.96 in the present study.

#### 2.2.2 Measurements of NSSI and suicide attempt

##### 2.2.2.1 NSSI

The Chinese version of the Functional Assessment of Self-Mutilation (C-FASM) was used to assess for method, frequency, and purpose of NSSI in the past 12 months preceding the survey (34). Participants were asked, “During the past 12 months, have you ever harmed yourself in a way that was deliberate but not intended to take your life?.” A list of eight NSSI methods was specified: hitting, head banging, stabbing, pinching, biting, scratching, burning, and cutting. For those who confirmed that they had engaged in NSSI, the frequency of NSSI was investigated. In the present study, NSSI was dichotomized (frequency of NSSI of three or more vs. fewer than three as yes or no, respectively) for analysis (35). The internal consistency reliability of C-FASM was 0.80 in the present study.

##### 2.2.2.2 Suicide attempt

Suicide attempt was measured using an item derived from the Global School-Based Student Health Survey (36). SA was defined as “once or more” in response to the question, “During the past 12 months, did you ever seriously consider attempting suicide?.”

### 2.3 Statistical analysis

Data analysis was performed from November 1, 2021, to April 1, 2022. Frequencies and proportions for categorical variables or mean (SD) for continuous variables were used to describe the characteristics of the participants and NSSI or SA among the participants by different variables. χ^2^ tests or two-tailed unpaired *t*-tests were used to compare the distribution between NSSI and non-NSSI or SA and non-SA participants according to different variables.

To examine the associations of depressive and anxiety symptoms with NSSI and SA, we used unconditional logistic regression models to estimate the unadjusted and adjusted odds ratios (OR)s and 95% CI of NSSI and SA by depressive and anxiety symptoms separately for participants who engaged in NSSI and SA. In the adjusted models, we adjusted for regional areas, ethnicity, gender, single child, family structure, caregiver’s educational level, living environment, academic pressure, emotional management ability (continuous data) and social support (continuous data).

We conducted subgroup analysis to examine whether there were any gender differences in the associations *via* calculating a ratio of ORs as we did in our previous study (37).

**FIGURE 1:**
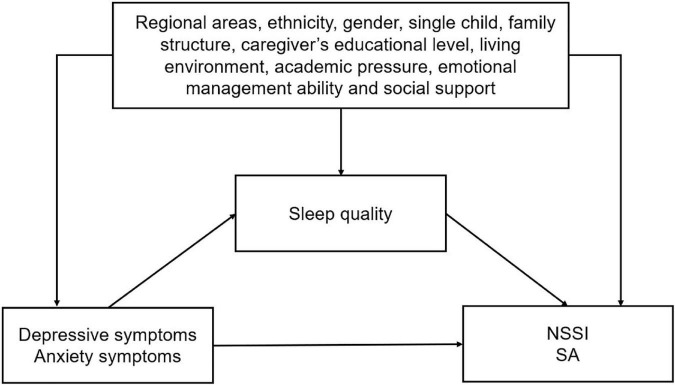
Proposed theoretical model.

To examine the assumption of the mediated effect of sleep quality on the association of depressive and anxiety symptoms with NSSI and SA, we conducted mediation analyses by a logistic decomposition of the total effects into direct and indirect effects using the “ldecomp” command in the Stata (38). Figure 1 shows the theoretical framework underlying our mediation analysis.

Data were missing in ethnicity (7.8%), single child (0.1%), family structure (0.5%), caregiver’s educational level (2.1%), living environment (3.0%), academic pressure (1.7%), and emotional management ability (0.4%). We imputed these missing covariates by using the monotone logistic regression method based on other sociodemographic covariates by creating 5 imputed datasets (39). The threshold of significance was defined as *P* < 0.05. All tests were two-sided unless otherwise specified, and all analyses were conducted using IBM SPSS Statistics, version 25.0 (IBM Corp., Armonk, NY, USA) and Stata (version 14.0, Stata Corp LLC, College Station, TX, USA).

## 3 Results

### 3.1 Participant characteristics

Of 1,844 students, 40 did not provide the consent form, 28 were absent from school on the day of the survey, and 5 submitted an incomplete questionnaire with at least 15% of the items unanswered. The final sample included 1,771 (94.0%) students. Of 1,771 participants, 994 were boys (56.1%) and 777 were girls (43.9%); 454 (25.6%) were recruited from Hengyang, 243 (13.7%) from Shenzhen, and 1,074 (60.6%) from Zhongshan city. The age of the participants ranged from 11 to 16 years, with the mean (SD) age of 12.9 (0.6) years 1,710 (96.6%) were Han ethnicity. The 12-month prevalence of NSSI among the participants was 17.1% (303 participants) and SA was 8.3% (147 participants). Participants who engaged NSSI three times or more or had SA in the 12 months preceding the survey were more likely to be girls; from single-parent family or recombine/cross-generation family; live in a poor environment; have heavy academic pressure; have poor emotional management ability; have poor social support; or have poor sleep quality. Other characteristics according to NSSI or SA were summarized in Table 1.

**TABLE 1:** Characteristics of participants by NSSI or SA.

Variables		NSSI		SA	
	**Total (*n* = 1771)**	**≥3 (*n* = 303)**	**χ^2^/*t***	**Yes (*n* = 147)**	**χ^2^/*t***
**Regional areas (*n*, %)**					
Hengyang	454 (25.6)	95 (31.4)		49 (33.3)	
Shenzhen	243 (13.7)	60 (19.8)	22.90***	36 (24.5)	26.27***
Zhongshan	1074 (60.6)	148 (48.8)		62 (42.2)	
**Ethnicity (*n*, %)**					
Han	1575 (88.9)	265 (87.5)	0.07	130 (88.4)	0.32
Others	58 (3.3)	9 (3.0)		6 (4.1)	
Missing	138 (7.8)	2 9 (9.6)		11 (7.5)	
**Gender (*n*, %)**					
Boys	994 (56.1)	125 (41.3)	32.83***	56 (38.1)	21.17***
Girls	777 (43.9)	178 (58.7)		91 (61.9)	
**Single child (*n*, %)**	301 (17.0)	44 (14.5)	1.59	19 (12.9)	1.88
**Family structure (*n*, %)**					
Core/joint family	1521 (85.9)	246 (81.7)		109 (76.2)	
Single parent family	95 (5.4)	21 (7.0)	6.55*	12 (8.4)	13.90**
Recombine/cross-generation family	146 (8.2)	34 (11.3)		22 (15.4)	
Missing	9 (0.5)	2 (0.6)		4 (2.7)	
**Caregiver’s educational level (*n*, %)**					
Junior high school or below	1041 (58.8)	180 (60.4)		90 (62.1)	
Senior high school	409 (23.1)	77 (25.8)	2.29	36 (24.8)	1.25
College or above	284 (16.0)	41 (13.8)		19 (13.1)	
Missing	37 (2.1)	5 (1.7)		2 (1.4)	
**Living environment (*n*, %)**					
Good	1147 (64.8)	165 (56.3)	17.53***	71 (51.1)	16.81***
Moderate or poor	570 (32.2)	128 (43.7)		68 (48.9)	
Missing	54 (3.0)	10 (3.3)		8 (5.4)	
**Academic pressure (*n*, %)**					
Heavy or more	816 (46.1)	173 (57.9)	17.509***	89 (61.8)	14.06***
Moderate or less	925 (52.2)	126 (42.1)		55 (38.2)	
Missing	30 (1.7)	4 (1.3)		3 (2.0)	
**Emotional management ability (M, SD)^a^**	11.4 ± 3.2	9.2 ± 3.1	13.63***	8.5 ± 3.0	12.11***
**Social support (M, SD)**	67.2 ± 15.9	57.8 ± 18.4	10.08***	53.7 ± 18.2	9.56***
**Depressive symptoms (*n*, %)**	357 (20.2)	151 (49.8)	200.03***	99 (67.3)	221.79***
**Anxiety symptoms (*n*, %)**	548 (30.9)	202 (66.7)	218.31***	114 (77.6)	163.00***
**Sleep quality** (***n*, %)**					
Good	1135 (64.3)	114 (37.7)	112.00***	42 (29.0)	86.00***
Poor	630 (35.7)	188 (62.3)		103 (71.0)	
Missing	6 (0.3)	1 (0.3)		2 (1.3)	

^a^Seven individuals with emotional management ability missing. *P < 0.05; **P < 0.01; and ***P < 0.001.

### 3.2 Association of depression and anxiety with NSSI

The prevalence of depressive and anxiety symptoms among the overall sample was 20.2% (357 participants) and 30.9% (548 participants), respectively. The prevalence of NSSI was 42.3% (151 participants) among the participants with depressive symptoms, and 36.9% (202 participants) among the participants with anxiety symptoms. Participants who had depressive or anxiety symptoms reported an increased risk of NSSI (Table 2). Significant associations were found between depressive or anxiety symptoms and NSSI in both unadjusted and adjusted models. In the adjusted model (model 2 in Table 2) for NSSI, the aOR was 1.89 (95% CI 1.34–2.65) for depressive symptoms and 2.84 (2.05–3.94) for anxiety symptoms. In the subgroup analyses, significant associations of depressive and anxiety symptoms with NSSI were found in both boys and girls, except for the association between depressive symptoms and NSSI in boys; the aOR was 1.55 (95% CI 0.92–2.58) (Table 3). However, no significant differences between the genders were found in the associations of depression or anxiety with NSSI (all *P* > 0.05).

**TABLE 2:** Frequencies, prevalence, and odds ratio of depressive and anxiety symptoms by NSSI or SA.

**Variables**		Unadjusted		Adjusted^a^	
	***n* (%)**	**OR (95% CI)**	***P***	**OR (95% CI)**	***P***
**NSSI**					
**Depressive symptoms**					
No (*n* = 1474)	17 (11.5)	1.00 (reference)		1.00 (reference)	
Yes (*n* = 357)	151 (42.3)	6.09 (4.65–7.97)	<0.001	1.89 (1.34–2.65)	<0.001
**Anxiety symptoms**					
No (*n* = 1223)	101 (8.3)	1.00 (reference)		1.00 (reference)	
Yes (*n* = 548)	202 (36.9)	6.49 (4.96-8.48)	<0.001	2.84 2.05-3.94)	<0.001
**SA**					
**Depressive symptoms**					
No (*n* = 1474)	57 (3.9)	1.00 (reference)		1.00 (reference)	
Yes (*n* = 357)	99 (27.7)	10.92 (7.55-15.80)	<0.001	3.20 (2.03–5.05)	<0.001
**Anxiety symptoms**					
No (*n* = 1223)	33 (2.7)	1.00 (reference)		1.00 (reference)	
Yes (*n* = 548)	114 (20.8)	9.47 (6.33-14.17)	<0.001	2.98 (1.84–4.84)	<0.001

^a^Adjusted for regional areas, ethnicity, gender, single child, family structure, caregiver’s educational level, living environment, academic pressure, emotional management ability and social support.

**TABLE 3:** Frequencies, prevalence, and odds ratio of depressive and anxiety symptoms by NSSI or SA in girls and boys, and the gender ratio.

**Variables**	Boys (*n* = 994)			Girls (*n* = 777)			Ratio of the odds ratio in boys versus girls	
	*n* (%)	Adjusted OR^a^ (95% CI)	*P*	*n* (%)	Adjusted OR^a^ (95% CI)	*P*	Ratio of two odds ratios (ROR)	One-sided *P*-value
**NSSI**				**NSSI**				
**Depressive symptoms**								
No	74 (8.9)	1.00 (reference)		78 (13.4)	1.00 (reference)			
Yes	51 (31.5)	1.61 (0.97–2.66)	0.067	100 (51.2)	2.06 (1.29–3.30)	0.003	0.78	0.242
**Anxiety symptoms**								
No	49 (6.8)	1.00 (reference)		52 (10.3)	1.00 (reference)			
Yes	76 (27.3)	2.42 (1.50–3.89)	<0.001	126 (46.7)	3.23 (2.07–5.05)	<0.001	1.09	0.403
**SA**				**SA**				
No	23 (2.8)	1.00 (reference)		25 (4.3)	1.00 (reference)			
Yes	33 (20.4)	2.54 (1.27–5.08)	0.009	66 (33.8)	3.52 (1.89–6.56)	<0.001	0.72	0.246
**Anxiety symptoms**								
No	13 (1.8)	1.00 (reference)		20 (3.9)	1.00 (reference)			
Yes	43 (15.4)	3.83 (1.79–8.21)	0.001	71 (26.3)	2.55 (1.34–4.86)	0.004	1.50	0.212

^a^Adjusted for regional areas, ethnicity, single child, family structure, caregiver’s educational level, living environment, academic pressure, emotional management ability and social support.

### 3.3 Association of depression and anxiety with SA

The prevalence of SA was 27.7% among the participants with depressive symptoms, was 20.8% among the participants with anxiety symptoms. Participants who had depressive or anxiety symptoms also reported an increased risk of SA (Table 2). Significant associations were found between depressive or anxiety symptoms and SA in both unadjusted and adjusted models. In the adjusted model (model 2 in Table 2), the aOR was 3.20 (95% CI 2.03–5.05) for depressive symptoms and 2.98 (95% CI 1.84–4.84) for anxiety symptoms. Significant associations were also found both in boys and girls (Table 3). However, no gender differences were found in these associations (all *P* > 0.05).

### 3.4 Mediation analysis

**FIGURE 2:**
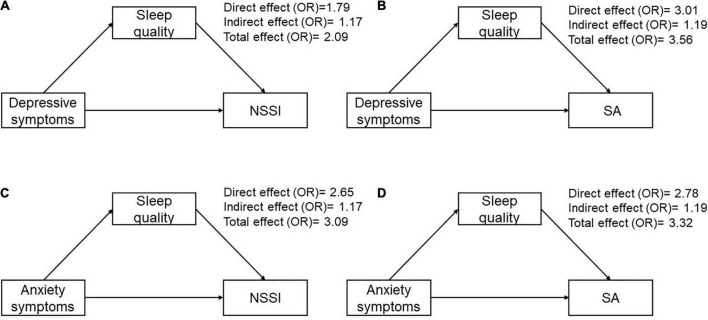
The odds ratio of proposed theoretical model. **(A)** Direct and indirect effects mediated through sleep quality of depressive symptoms on NSSI. **(B)** Direct and indirect effects mediated through sleep quality of depressive symptoms on SA. **(C)** Direct and indirect effects mediated through sleep quality of anxiety symptoms on NSSI. **(D)** Direct and indirect effects mediated through sleep quality of anxiety symptoms on SA.

The results from the mediation analysis indicated a significant direct effect of depressive and anxiety symptoms on NSSI and SA. The indirect effect through sleep quality on NSSI was 1.17 (95% CI 1.02–1.34) for depressive symptoms, and 1.17 (95% CI 1.01–1.34) for anxiety symptoms, with the mediating ratio of 21.1 and 13.9%, respectively. The indirect effect through sleep quality on SA was 1.19 (95% CI 1.01–1.42) for depressive symptoms, and 1.19 (95% CI 1.00–1.42) for anxiety symptoms (Table 4 and Figure 2), with the mediating ratio of 13.6 and 14.7%, respectively.

**TABLE 4:** Logistic regression model of direct and indirect effects through sleep quality of depressive and anxiety symptoms on NSSI and SA.

	NSSI		SA	
Effects	OR (95% CI)^a^	*P*	OR (95% CI)^a^	*P*
Total effect of depression	2.09 (1.55–2.81)	<0.001	3.56 (2.14–6.04)	<0.001
Direct effect of depression	1.79 (1.31–2.43)	<0.001	3.01 (1.80–5.03)	<0.001
Indirect effect through sleep quality	1.17 (1.02–1.34)	0.030	1.19 (1.01–1.42)	0.041
Mediating ratio (%)	21.1		13.9	
Total effect of anxiety	3.09 (2.25–4.26)	<0.001	3.32 (2.02–5.44)	<0.001
Direct effect of anxiety	2.65 (1.93–3.65)	<0.001	2.78 (1.73–4.46)	<0.001
Indirect effect through sleep quality	1.17 (1.01–1.34)	0.035	1.19 (1.00–1.42)	0.045
Mediating ratio (%)	13.6		14.7	

^a^Adjusted for regional areas, ethnicity, gender, single child, family structure, caregiver’s educational level, living environment, academic pressure, emotional management ability, social support.

## 4 Discussion

In this cross-sectional study, we found that depressive and anxiety symptoms were independently associated with NSSI and SA. No significant gender differences in these associations of depressive and anxiety symptoms with NSSI and SA were observed. The mediation proportion of sleep quality on the association of depressive and anxiety symptoms with NSSI, as well as depressive and anxiety symptoms with SA were 21.1, 13.9, 13.6, and 14.7%, respectively.

### 4.1 The prevalence of depressive and anxiety symptoms, NSSI and SA

In the present study, we found that the prevalence of depressive and anxiety symptoms was 20.2 and 30.9%, respectively, higher than that reported in previous studies (40). This may be due to the fact that the present study was conducted during the COVID-19 pandemic, and depressive and anxiety symptoms were sensitive to traumatic events and their social and economic consequence (41). As an event that can cause physical, emotional, and psychological harm, the COVID-19 pandemic and the policies developed to prevent it spread can be considered stressors and disrupted daily living for most people all over the world (42). Of note, the prevalence of depressive and anxiety symptoms in the present study was lower than in those studies that were conducted at early stage of the COVID-19 pandemic (43), which implied people were adapting to new environment with stressors. In the present study, the 12-month prevalence of NSSI and SA was 17.1 and 8.3%, respectively, which was consistent with some previous studies (2, 44), but inconsistent with another (45). The inconsistent prevalence among previous studies may be attributable to different measurements and criteria, apart from study population and study timing (45, 46).

### 4.2 Association of depressive and anxiety symptoms with NSSI and SA

Previous studies have shown a consistent association of depressive symptoms with NSSI and SA among children and adolescents (10, 13). A cross-section study of 1,674 Korean high students found that depressive symptoms were independently associated with NSSI (14). A prospective study of 506 Swedish adolescents found depressive symptoms in baseline predict the increased risk of NSSI 1 year later, although subsequent follow-up failed to find the direction of the association between depressive symptoms and NSSI (19). Our study has extended this literature by addressing the limitation of the lack of equivalent research within Chinese community adolescent populations, and the findings were consistent with other studies conducted in clinical populations (13, 15).

Compared with the studies that examined the association of depressive symptoms with NSSI and SA, there were less studies that examined the association of anxiety symptoms with NSSI and SA, and the findings were mixed. Some studies indicated that anxiety symptoms were independently associated with NSSI and SA (10, 47), others indicated null association (13, 14). This discrepancy may be related to study population, epidemiologic characteristics of anxiety symptoms, NSSI and SA, and the controlling variables. In the present study, we recruited a representative community adolescent population with comparable prevalence of anxiety symptoms, NSSI and SA, and adjusted for up to 12 potential confounders, suggesting that anxiety symptoms were independently associated with NSSI and SA among adolescent at early adolescence, which consistent with most previous studies conducted in western countries (47). Individuals suffering with anxiety, worry, and fear may seek escape from their suffering by NSSI or SA, thus, it is reasonable that anxiety symptoms were independently associated with NSSI and SA.

Previous study suggested that the tradition of son preference in China, may contribute to the poorer mental health outcomes (including depressive symptom, anxiety symptom and low self-esteem) previously observed in Chinese female (35). Yet no study examined gender differences in the associations between depressive and anxiety symptoms with NSSI and SA among Chinese community-based adolescent population. Although there was no significant association of depressive symptom with NSSI in boys, our study suggested that there were no gender differences in association of depressive and anxiety symptoms with NSSI and SA, which in line with previous studies conducted in other populations (18, 19). There are several possible explanations for the findings. Firstly, girls with depressive and anxiety symptoms may more likely than boys to seek help from friends, family, and health services. Secondly, it is possible that depressive symptoms are more stigmatized in boys than in girls, so boys may be less likely to report symptoms (18). However, evidence for this latter explanation was mixed (48). Further studies are needed to ascertain whether there are gender differences in the associations of depressive and anxiety symptoms with NSSI and SA.

### 4.3 Mediating effect of sleep quality on association of depressive and anxiety symptoms with NSSI and SA

To our knowledge, this is the first study to estimate the mediating effect of sleep quality on the association of depressive and anxiety symptoms with NSSI and SA. We found the mediating proportion of sleep quality on association of depressive symptoms with NSSI and SA, as well as anxiety symptoms with NSSI and SA were 21.1, 13.9, 13.6, and 17.4%, respectively. The associations between depressive symptom, anxiety symptom, sleep quality, and NSSI and SA are complex. A longitudinal study of 1,457 Swedish adolescents found that depressive symptoms could explain why insomnia was a risk for NSSI (49). Liu and colleagues also found that the association between frequent nightmares and NSSI could mediate through depressive symptoms (50). In contrast, a study of 127 adults with psychiatric disorders suggested that the effect of current major depression on suicidal ideation could mediate through sleep disturbance (51). A study conducted in Chinese adolescents suggested that sleep quality plays a mediation role on the association between emotional and behavioral problems and suicidal ideation (23). The association between depressive symptoms, anxiety symptoms, emotional behavioral problems, and sleep quality may be bi-directions or co-occur, thus both hypothesized that there are mediating effects of depressive symptoms, anxiety symptoms, or sleep quality on these associations are reasonable. This finding implied that screening adolescents for poor sleep quality may help identify those at risk of NSSI and SA, improving sleep quality could mitigate the association of depressive and anxiety symptoms with NSSI and SA. Further studies are needed to ascertain the directions of these associations.

### 4.4 Strengths and limitations

Although there were studies examined the association of depressive symptoms, suicidal behaviors, and sleep problems (21, 50), this study is the first to examine the association of depressive and anxiety symptoms with NSSI and SA among Chinese adolescents, which also examined the mediation role of sleep quality therein. The study population focused on early adolescence when the onset of NSSI and suicidality usually occur, which may contribute to better understand the proximal risk factors of NSSI and SA. The mediation analyses provide evidence on how depressive and anxiety symptoms associated with NSSI and SA, which is helpful for developing potential interventions.

Several limitations should be noted. Firstly, the cross-sectional study design makes it impossible to ascertain the causal association between depressive symptoms, anxiety symptoms, sleep quality and NSSI and SA. Nonetheless, our findings pertaining to the association of depressive symptoms, anxiety symptoms, sleep quality with NSSI and SA were similar to this in prospective studies (19, 47, 52). A prospective longitudinal study is benefit for clarifying the direction of association between depressive symptoms, anxiety symptoms and sleep quality. Secondly, all the variables were measured by a self-reported questionnaire, which may augment underestimation of some sensitive issues regarding depressive symptoms, anxiety symptoms as well as NSSI and SA and increase potential reporting bias and recall bias. This may ultimately influence the strength of the observed associations and our results may represent a more conservative estimation than was truly presented. However, the prevalence of depressive symptoms, anxiety symptoms, NSSI and SA were comparable with previous studies that conducted during the COVID-19 pandemic (41, 53), and previous study demonstrated that the method of information gathering from a young person and school-based students regarding self-harm and risk factors is likely to be reliable (54). Thirdly, we only used the cut-off scores for depressive and anxiety symptoms assessment, which is inconsistent with the DSM-5-dimensional approach, although it is consistent with the DSM- IV categorical approach. In addition, anxiety symptoms are very broad, and the GAD only applies to generalized anxiety, thus anxiety symptoms assessed in the present study referred to generalized anxiety. Thus, in the future studies, dimensional approach assessment with more comprehensive tools for depressive and anxiety symptoms are needed. Fourthly, the study focused on seventh-grade adolescents, therefore, findings of this study may not be generalized to adolescents who were at other study stages, since variables assessment in present study may change with age increase. Finally, although we have adjusted for many important confounding factors, potential confounding bias owing to unmeasured factors such as rumination, impulsivity, could not be ruled out. Some physical problems, such as chronic pain, that could affect sleep quality were not excluded in this study. Overall, caution should be exercised when applying the results to the total population of Chinese adolescents.

## 5 Conclusion

Independent associations of depressive and anxiety symptoms with NSSI and SA were observed in Chinese adolescents, and there were no significant gender differences in these associations. Moreover, sleep quality paly a mediating role in the associations of depressive and anxiety symptoms with NSSI and SA. These findings extending existing literature by further exploring the association of depressive symptoms, anxiety symptoms, sleep quality, with NSSI and SA. Targeted interventions for adolescents’ NSSI and SA should focus on those who have depressive and anxiety symptoms, and poor sleep quality.

## Data availability statement

The raw data supporting the conclusions of this article will be made available by the authors, without undue reservation.

## Ethics statement

The studies involving human participants were reviewed and approved by the Institutional Review Board of Guangzhou Medical University. Written informed consent to participate in this study was provided by the participants’ legal guardian/next of kin.

## Author contributions

TJ and JT participated in the design of the study and performed the statistical analysis. TJ drafted the manuscript. SG, YZ, YL, and XX participated in the data collection. YY and JT participated in the design and coordination. RC and YY revised the manuscript and help to analysis the data. All authors read and approved the final manuscript.
